# Sustaining the National Spinal Cord Injury Registry of Iran (NSCIR-IR) in a Regional Center: Challenges and Solutions

**Published:** 2020-04

**Authors:** Zahra AZADMANJIR, Zahra MOHTASHAM-AMIRI, Seyed-Mahdi ZIABARI, Leila KOCHAKINEJAD, Hamid HAIDARI, Mina MOHSENI, Hadis SABOUR, Zahra KHAZAEIPOUR, Mahdi SHARIF-ALHOSEINI, Zahra GHODSI, Abbas AMIRJAMSHIDI, Farshad AKBARZADEH, Kazem ZENDEHDEL, Amir AZARHOMAYOUN, Khatereh NAGHDI, Gerard OREILLY, Ellen MERETE, Alexander R VACCARO, Edward C BENZEL, Seyed Behzad JAZAYERI, Vafa RAHIMI-MOVAGHAR

**Affiliations:** 1. Sina Trauma and Surgery Research Center, Tehran University of Medical Sciences, Tehran, Iran; 2. Department of Health Information Management, School of Allied Medical Sciences, Tehran University of Medical Sciences, Tehran, Iran; 3. National Inistitue for Medical Research Development (NIMAD), Tehran, Iran; 4. Guilan Road Trauma Research Center (GRTRC), Guilan University of Medical Sciences, Rasht, Iran; 5. Brain and Spinal Cord Injury Research Center, Neurosciences Institute, Tehran University of Medical Sciences, Tehran, Iran; 6. Department of Neurosurgery, Sina Hospital, Tehran University of Medical Sciences, Tehran, Iran; 7. Cancer Research Center, Cancer Institute, Tehran University of Medical Sciences, Tehran, Iran; 8. Department of Epidemiology and Preventive Medicine, School of Public Health and Preventive Medicine, Monash University, Melbourne, Australia; 9. Autonomic Unit, National Hospital for Neurology & Neurosurgery, Queen Square, London, UK; 10. Institute of Neurology, University College London, London, UK; 11. Department of Orthopaedic Surgery, The Rothman Institute, Thomas Jefferson University, Philadelphia, USA; 12. Cleveland Clinic Foundation, Department of Neurosurgery, Cleveland, Ohio, USA; 13. Department of Urology, University of Florida, Jacksonville, FL, USA

**Keywords:** Trauma, Spinal cord, Disease registries, Iran

## Abstract

**Background:**

The National Traumatic Spinal Cord Injury Registry in Iran (NSCIR-IR), was implemented initially in three hospitals as a pilot phase from 11 Oct 2015 to 19 Jun 2016 and has been active in eight centers from 19 Jun 2016. Poursina Hospital, a trauma care referral center in Rasht, Guilan Province of Iran is one of the registry sites, and has been involved in registering eligible patients since 1 Jan 2016. This study aimed to identify the challenges and solutions for sustaining the NSCIR-IR in a regional center.

**Methods:**

This was a mixed-methods study. For the quantitative analysis, a retrospective observational design was used to measure case capture or case identification rate, mapping cases in the registry against those eligible for registry inclusion amongst the register of hospital admissions. For the qualitative component, data was collected using focus group discussions and semi-structured interviews, followed by thematic analysis.

**Results:**

From 19 Jun 2016 to 24 Jan 2018, the proportion of case capture (case identification rate) was 17%. The median time between case identification and data entry to the system was 30.5 d (range: 2 to 193 d). Thematic analysis identified a lack of trained human resources as the most important cause of low case identification rate and delay in data completion.

**Conclusion:**

Recruitment and education to increase trained human resources are needed to improve case capture, the timeliness of data input and registry sustainability in a regional participating site.

## Introduction

Poursina teaching hospital is a referral care hospital for acute trauma in Rasht, in the center of Guilan Province in the north of Iran. The north of Iran is a tourism and frontier region and it is located between the Caspian Sea and Alborz mountains. Due to the weather condition, the probability of road traffic crashes is extremely high in this part of Iran. The province area is 14,042 km^2^, and its population was 2.530,696 in 2016 (1). The size of Poursina hospital is approximately 36,000 m^2^ and includes clinical departments for orthopedics, general surgery, reconstructive surgery, neurosurgery, neurology, emergency, a general intensive care unit (ICU) and neurosurgery ICU, operating rooms, rehabilitation therapists and a special poly-clinic for outpatient care (2). According to a study, 3,598 patients with injuries were admitted between Sep 2005 and Jul 2006. Of these, 73.8% were related to traffic crashes and 15.7 % related to falls (3). In 2016, of 210,000 patients admitted to the Poursina hospital, 37,200 patients were hospitalized, 60% of which were trauma patients. Hence, Poursina hospital was the first center for collaborating actively in National Spinal Cord/column Injury Registry of Iran (NSCIR-IR).

NSCIR-IR, the national hospital-based registry of Traumatic Spine Fracture (TSF) with or without Traumatic Spinal Cord Injury (TSCI), was designed during the period from Nov 2014 to Oct 2015. It was then implemented in three hospitals as a pilot phase for eight months (11 Oct 2015 to 19 Jun 2016) and is running currently at eight centers: one center in each of Rasht, Shiraz, Tabriz, Orumieh, Yazd, Kashan and two centers in Tehran (4). The workflow in the NSCIR-IR started with case finding by the registrar according to the defined inclusion and exclusion criteria and required data recorded in both paper and electronic case report forms. Data could be directly entered into the NSCIR-IR software and the submitted data were transferred to a quality reviewer for checking completeness, accuracy and consistency of the information. There was additional checking for the fracture type of spinal vertebra, for which the quality reviewer observed the CT, MRI and XRay for each patient. If the type of fracture or any other items seemed to be inaccurate, the feedback was given to the registrar for correction.

Poursina hospital has been involved in registering eligible TSF/TSCI patients since 1 Jan 2016. However, the following three issues were considered to be most important for the sustainability of this regional center as a registry site: (i) case identification rate (ii) data accuracy and (iii) delay in completing data by the registrar.

Therefore, it was essential to conduct an audit of registry at the Poursina hospital site to monitor the case identification rate, accuracy, timeliness and to determine the causes of, and potential solutions for, any identified issues.

## Materials and Methods

**Fig. 1:**
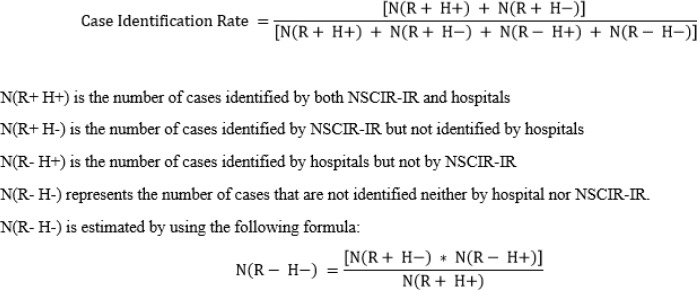
Case Identification Rate Formula

To determine the case identification rate, we compared the statistics and the list of patients identified by the registry (registered in the NSCIR-IR software) with the list of available patients admitted to the hospital. The list was extracted through a search in the hospital information system (HIS) based on ICD-10 codes. The codes of eligible patients were S14.0–S14.1, S24.0–S24.1, S34.0–S34.1 for SCI and S12.0–S12.1, S12.7, S22.0–S22.1, S32.0–S32.2 for spine fractures. We used the formula mentioned in our previous study to estimate the case identification rate (Fig. 1) (5).

To determine the registry timeliness, we calculated the time between the identification (admission date) and the date of data entry into the software for each patient registered in NSCIR-IR software from 19 Jun 2016 to 24 Jan 2018.

The registry headquarters team held the focus group discussion meeting in Gilan in Feb 2018. There was no sampling to select people. Members of the focus group discussion were all of active participants in the Poursina regional registry center and registry headquarters. The meeting included 12 people with the specializations of neurosurgery, nursing, community medicine, health services management, information technology, health information management, emergency medicine, and a researcher in the field of trauma.

The meeting lasted six hours and several tasks were performed: at first, we arranged an unstructured interview with the registrar. The A one-hour interview was recorded and responses labeled with key terms which were the main themes of the discussion. Labels were communication, human resources, feedback, motivation, software, case report form infrastructure, and education. Second, the team extracted statistics of admitted eligible patients over a pre-specified time period through the hospital information system (HIS) with the cooperation of the medical coding unit and a number of patient records were reviewed at random. In the end, identified problems were grouped on the base of the cause. Then we discussed problems with managers in the research center. We used the Ishikawa diagram or cause-and-effect diagram (also known a Fishbone) to categorize the cause of the problem.

The Ethics Committee of the Sina Trauma and Surgery Research Center, Tehran University of Medical Sciences, which is the coordinating unit of the NSCIR-IR, approved this study.

## Results

As Table 1 shows, case identification rate was low in both pilot phase and implementation phase (16% in pilot phase and 17% in implementation phase). In the implementation phase, 77 new patients (with TSF with or without SCI) were registered in the NSCIR-IR software that 45 of them were not available at the hospital coded patient list in HIS. However, there were 295 eligible patients in the HIS list that 263 of them were not recorded in NSCIR-IR software. Specially, there were eight patients with SCI admitted to the hospital from 19 Jun 2016 to 24 Jan 2018, according to the hospital medical coding unit (HIS list). After searching related data in the NSCIR-IR software, we identified one of those eight patients with SCI, in the registry. In contrast, 12 SCI patients detected by the registry were not in the obtained HIS list. According to the hospital’s clinical coder, on average the 20% medical records related to hospital discharged patients during 2016 had not been coded due to lack of human resources. Comparison of recorded data in the NSCIR-IR system, the information correctness was observed for about 77 hospital medical records of patients. Birth data were recorded on the basis of the patient’s identification documents not the hospital information system, except in a particular case where the day and month of the patient’s admission date were recorded as the day and month of the patient’s birth date, which was due to the hospital information system error.

**Table 1:** Comparison of the number of eligible cases identified by the registry with available in the hospital

***Phase ***	***Total eligible case admitted to the hospital***	***Detected by NSCIR-IR Registry***					***Detected by Registry/Unavailable in Hospital Patients List***	***Common between Hospital & Registry***	***Available in Hospital/not Detected by Registry***	***Detected from HIS (in the hospital list)***	***Case Identification Rate***
		**Quality Verified**	**Under Quality review**	**Completing/Need to edit**	**Rejected**	**Total recorded in the registry**					
Pilot phase ^1^	131	14	0	18	0	32	12	20	99	119	16%
Implementation phase^2^	340	36	2	38	1	77	45	32	263	295	17%

1 Evaluation date was 19 June 2016

2 Evaluation date was 24 January 2018

According to Table 2, there was a significant delay in completing data entry to the case report forms which were available in the software and correcting the data needed by edit after quality review. There were 20 records of SCI patients in the NSCIR-IR software where were not completed or corrected at the time of the visit. Data of 74 identified cases (64%) from 2016–2017 were not verified. Moreover, the last time for registration of a case was Jul 2017. The mean and median of time from case identification to registration into the software for 77 patients were 52 and 30.5 d respectively.

**Table 2:** Total registration by Poursina hospital in the registry (1 Jan 2016–24 January 2018)

***Status N (%)***	***Completing/open record 9 (7.75%)***	***Need to correction 45 (38.79%)***	***Under Quality review 2(1.72%)***	***Rejected 2 (1.72%)***	***Quality Verified 58 (50%)***	***Total registration 116(100%)***
2016	7	17	1	2	46	73
2017	2	28	1	0	12	43
2018	0	0	0	0	0	0

**Fig. 2:**
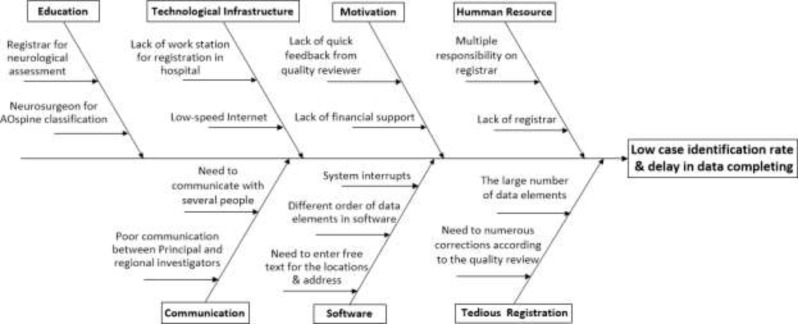
Ishikawa diagram for cases of low case identification rate and delay in data completing

The result of the interview with registrar illustrated possible factors which contributed to the low identification rate and delay in data completing. These factors are represented in Fig. 2 in the form of an Ishikawa diagram.

Potential solutions were discussed and agreed on for each problem after recognition of them. The potential solutions to each of the problems are presented in Table 3.

**Table 3:** Identified Problems obtained from interview with registrar and related solutions

***Problem category***	***Problem***	***Solution***
A. Human Resource	Lack of registrar Multiple responsibility of registrar for care and research	Providing of at least two full-time registrars
B. Tedious Registration	The large number of data elements Need to have numerous corrections according to the quality review	Promotion in CRFs: reordering of data elements
C. Motivation	Lack of financial support Lack of quick feedback from quality reviewer Limited access of quality reviewer to the hospital Picture Archiving and Communication System (PACS)	Provide financial support directly to the registrar (per case) Facilitating access to PACS
D. NSCIR-IR Software	Different order of data elements in electronic forms System interrupts and bugs Need to enter free texts for the location of the injury and the place of living	Promotion in software
E. Technological Infrastructure	Low-speed internet in hospital Lack of work station for registration in hospital	Assignment of equipped workstation to registry
F. Communication	Need to communicate with several people Poor communication between principal and regional investigators	Strengthen communication between registry headquarters and regional center
G. Training	Insufficient education to registrar for neurological assessment Lack of education to hospital Neurosurgeon for AOspine classification	More training courses

## Discussion

This study employed mixed-methods (quantitative and qualitative) to evaluate the NSCIR-IR, and identify the challenges to registry sustainability in one regional participating site. Specifically, case identification rate, data accuracy, and timeliness were measured, followed by the determination of potential causes and solutions for identified challenges to ensure data quality. Many eligible patients were admitted to the hospital but were not identified by the NSCIR-IR. Moreover, there were many identified cases by the NSCIR-IR but were not found in the hospital coded list. However, case identification rate should increase in the NSCIR-IR, but it was more than the USA SCI registry. In USA according to report of National Spinal Cord Injury Database, 13% of all new cases (from 10,000 new spinal cord injury each year) were included in the database each year(6).

Medical coding is essential for clinical data retrieval and accurate statistical evaluation in epidemiological studies and surveillance (7). Delays in the coding of the patient data leads to unavailability, inaccessibility of the information; data could not be recalled when needed (8). In a national cohort study to evaluate effect of Statin on reduction in mortality in patients with chronic obstructive pulmonary diseases, researchers could not know the cause of death in 44% patients due to delays in coding (9). Similarly, in our project, due to the delay in file coding, 45 patients were not on the hospital list. Therefore, timeliness is an important indicator for quality of coding. In Iran, all study participants emphasized on timeliness as an audit criterion in the medical coding audit model (10). The coding timeliness is difficult in Iran and one reason for delayed coding was the long process of record financial auditing in the accounting and income unit of hospitals. It leads to the late delivery of the patient records to the medical coding unit (11). In our study, there were a limited number of expert coders employed in hospital, especially given the high volume of medical records.

For the registry, we found that the main cause of the low identification rate of Poursina hospital was the shortage of manpower for data collection. The importance of manpower as a registrar is firmly established “registries are maintained by registrars” (12). Supplying human resources and maintain trying is considered as a fundamental issue for the project continuation. After recruiting full-time human resources, motivation is the second issue that needs to be addressed is increased motivation through financial support is a solution. Although budget is limited, increased financial support can be effective in boosting the incentive for cooperation. Researchers in the field of trauma registry believe that the maintenance cost of trauma registry is high and they should be given adequate budgets (12). Rapid, clear, transparent and appropriate feedback is another solution to maintain registrar motivation. According to our findings, the delay in providing feedback from quality reviewer to the registrar and simultaneously direct and clear transparent feedback to the executive manager and principal investigator (PI) was one of the reasons for the lower motivation. Feedback should be provided to registrars by up to two weeks from the time of data submission. If no feedback is sent during this period, data correction for the registrars will be difficult due to forgetfulness of patient status or patient discharge (13, 14). We found that our long-form leads to fatigue. Using less free-text data in the software can reduce the time of data entry, avoiding deletion of essential data.

The other problem was different arrangement of questions or data in a paper case report form (CRFs) with the designed forms in the software NSCIR-IR. In a stroke registry, similarity of the paper and electronic CRFs was mentioned as an advantage due to reducing error probability during transcription (15). Therefore, we need to reorganize our paper CRFs according to the order of data entry in the software.

Due to poor communication between principal investigator (PI) and regional registry center, more active ways of communication like telephoning should be used instead of sending a message or an email. Siegler recommended continuous periodic attendance meetings on the registry progress for better communication and cooperation of the team members (15).

## Conclusion

The examination of registry status at a regional participating site of NSCIR-IR helped to both identify problems in the regional center and improving the management and supervision of the registry. The information from this research is useful to improve the registration process in other centers and even other registries

## Ethical considerations

Ethical issues (Including plagiarism, informed consent, misconduct, data fabrication and/or falsification, double publication and/or submission, redundancy, etc.) have been completely observed by the authors.
